# 측정도구의 심리계량적 속성 1: 내용타당도

**DOI:** 10.4069/kjwhn.2021.01.31

**Published:** 2021-03-08

**Authors:** Eun-Hyun Lee

**Affiliations:** Graduate School of Public Health, Ajou University, Suwon, Korea; 아주대학교 보건대학원

## 서론

측정이란 규칙에 따라 사물, 사건 또는 현상에 숫자를 부여하는 것을 의미하며[1], 간호 현장에서 연구자나 실무자는 관심 개념을 직·간접으로 측정하게 된다. 예를 들어 체중은 체중기를 사용해서 직접적으로 측정하지만, ‘희망’ 같은 추상적인 개념은 간접적으로 측정하게 된다. 즉, 추상적 구성개념이 가지고 있는 속성을 측정가능한 지표(질문)로 만들어서 측정하는 것이다. 간호학에서 흔히 사용되는 간접적 측정방법은 자가보고형 도구(self-reported instrument)이다. 의학에서는 의료인이나 보호자가 아닌 환자가 자신의 건강상태를 주관적으로 평가하는 도구를 환자보고결과 측정도구(patient-reported outcome measure, PROM)라고 명명하고 있다[2]. 자가보고형 도구 또는 PROM 사용에서 가장 중요한 것은 심리계량적 속성(psychometric properties)이 만족되었는지 이다. 앞으로 본 학술지에서 심리계량적 측정속성에 대한 내용을 시리즈로 연재할 예정인데, 척도(scale), 검사(test), 도구(tool) 및 설문지(questionnaire)로 이름 붙여진 자가보고형 도구 또는 PROM 모두를 측정도구(instrument)라고 할 것이며, 그 첫 번째 측정속성으로 내용타당도에 대해 알아보고자 한다.

## 내용 타당도

내용타당도는 측정도구의 내용이 측정하고자 하는 구성개념을 얼마나 적절히 반영하고 있는가에 대한 것이다[3]. 내용타당도는 측정도구를 개발할 때 가장 먼저 수행해야 할 매우 중요한 부분으로, 크게 두 단계로 진행된다. Lynn [4]은 이를 개발단계와 판단-정량화(judgement-quantification) 단계로 나누었고, 이와 유사하게 Polit와 Yang [5]은 개념화 단계와 도구에 포함된 내용이 개념화된 구성개념을 잘 나타내는지에 대한 근거수립 단계로 분류하였다. 또한 COSMIN (COnsensus-based Standards for the selection of health Measurement INstruments) 연구에서는 새로운 도구 개발(PROM development) 단계와 개발된 도구의 관련성, 포괄성 및 이해력에 대한 평가 단계로 구분하였다[6]. 비록 내용타당도 수행 과정을 설명하기 위해 사용된 용어들은 조금씩 달랐지만, 그 내용은 거의 비슷하다고 할 수 있다. 즉, 첫 번째 단계는 측정하고자 하는 구성개념의 개념화를 포함한 예비측정도구 개발이라고 할 수 있으며, 두 번째 단계는 개발된 측정도구의 내용이 정의된 구성개념을 얼마나 반영하는지를 판단하는 단계라고 할 수 있다.

## 예비측정도구 개발 단계

측정도구 개발 단계에서 가장 먼저 해야 할 것은 측정하고자 하는 구성개념에 대한 정의(definition)를 확실하게 하는 것이다. 구성개념에 대한 정의를 내리기 위해서 사용하는 방법으로는 이론에 진술된 개념적 정의를 가져와 사용하거나, 문헌고찰을 통해 정의를 내리거나, 개념분석 및 질적연구를 통해 개념을 형성할 수 있다[7]. 이러한 과정에서 구성개념의 범위 및 개발된 측정도구를 사용할 대상자에 대한 사항도 확실하게 밝혀야 한다. 예를 들어, Lee 등[8]은 “건강관련 삶의 질” 측정도구 개발을 위한 내용타당도 개념화 과정에서 구성개념의 범위를 삶의 질보다 제한되지만 건강의 개념보다는 넓은 개념이라고 하였고, 측정도구를 사용할 대상이 “심혈관 질환자”임을 밝혔다.

개념적 정의가 규명되었으면, 그 다음으로는 구성개념이 가지고 있는 속성을 파악한다. 속성 도출을 위해 문헌고찰, 면담(일대일 인지적 면담 또는 포커스 그룹 면담), 질적연구 등을 통해 구성개념이 가진 속성을 파악할 수 있다. 이렇게 파악된 속성들을 이용해서 각 문항을 작성한다. 이때, 읽기 수준은 초등학교 6학년에서 중학교 1학년 정도이며, 문항은 모호하지 않게 기술해야 하고, 이중부정은 사용하지 않는 것이 좋다. 또한 전문용어나 외국어 사용은 피하는 것이 좋다[7]. 예를 들어, 암 환자 특이형 삶의 질 측정도구인 European Organization for Research and Treatment of Cancer Quality of Life Questionnaire, Core Module (EORTC QLQ C-30)의 초기 한글판에 “TV”라는 단어가 사용되었는데[9], 실제로 이 도구를 한국 암 환자에게 사용했던 연구에 의하면 저학력의 노인층 환자에서 영어 단어가 있었던 문항을 이해하는 데 문제가 있었던 것으로 나타났다[10].

이 외에도 측정도구 구성에 대해서도 고려해야 한다. 측정도구의 지시문(instruction), 회상기간(recall period) 및 응답에 대한 선택(response options) 등을 주의 깊게 결정해야 한다. 암환자를 대상으로 개발된 외상 후 성장 측정도구[11]를 보면, 지시문에 “…암 진단 이후 귀하의 경험과 일치하는 곳에 V 표시하여 주시기 바랍니다”라고 기술되어 있고 응답을 위한 특정한 회상기간은 제시되어 있지 않다. 대상자마다 암을 진단받은 기간이 다르고, 대상자들이 암 진단을 받은 시점부터 경험한 것을 회상해서 응답한다는 것은 쉽지 않다. 따라서 적절한 회상기간을 정해서 지시문에 기술해 주는 것이 측정오차 감소에 도움이 될 수 있다.

## 구성개념 반영에 대한 판단 단계

예비문항으로 이루어진 도구가 준비되었으면, 그 다음으로는 첫 번째 단계에서 정의된 구성개념에 대한 관련성(relevance), 포괄성(comprehensiveness) 및 이해력(comprehensibility)에 대한 평가가 이루어져야 한다. 가장 흔히 사용되는 방법은 전문 패널을 활용하는 것이다. 전문 패널에 필요한 인원에 대한 합의는 없지만, Lynn [4]은 5명 이상에서 10명이 바람직하다고 하였고, Waltz 등[12]은 20명까지도 추천하였다. 하지만 본 연구자의 경험에 의하면, 패널 전문가들이 측정하고자 하는 구성개념에 대해서 이론적으로 또한 실무적으로 얼마나 준비되어 있는지를 고려하는 것도 매우 중요하다.

Lawshe [13]는 전문가 내용타당도를 판단하기 위해 내용타당도 비율(content validity ratio, CVR)의 사용을 제안하였다. 전문가에게 문항이 얼마나 관련 있는지를 3점 척도에(필수적임, 사용 가능하지만 필수적인 것은 아님, 필요 없음) 응답하도록 요청하고, CVR=(ne-N/2)/(N/2)을 계산한다(ne, 필수적이라고 응답한 패널의 수; N, 총 패널의 수). CVR 값의 범위는 –1에서 +1이며, CVR의 임계치는 패널 전문가의 수에 따라 달라진다고 하였다. 예를 들어, 전문가 수가 5명, 10명일 때 각각 0.99, 0.62 이상이면 내용타당도를 만족한다고 보았다.

Lynn [4]이 제시한 내용타당도 지수(content validity index, CVI)는 간호학에서 가장 많이 사용하는 방법이다. 전문가 패널에게 문항이 얼마나 관련 있는지에 대해 4점 척도로(1점, 관련 없음; 2점, 수정을 하지 않는 한 관련 있다고 하기 어려움; 3점, 관련이 있으나 약간의 수정이 필요함; 4점, 매우 관련 있음) 답하도록 요청한다. 그리고 각 문항에 대해 3점 또는 4점에 답한 전문가의 비율인 CVI를 산출한다. 문항의 CVI (item-level CVI, I-CVI) 값은 전문가가 3–5명이면 1.00, 그리고 6–10명 이면 0.78 이상이어야 문항의 내용타당도가 만족된 것이라고 보았다. 전체 측정도구의 CVI (average of content validity index for scale, S-CVI/Ave) 산출은 I-CVI를 합해서 문항의 수로 나누어 산출하며, Polit와 Beck [14]은 0.90 이상을 권장하였다.

CVI는 일치 비율(portion agreement)에 대한 계산으로 간단하고 이해하기 쉽지만, “우연의 일치(chance agreement)”로 인한 과대 추정의 가능성을 배제할 수 없다. 따라서 Wynd 등[15]은 이를 보정하는 다중평가자 카파계수(multi-rater Kappa coefficient)를 CVI와 같이 사용할 것을 권장하였다. 하지만, 카파계수는 측정도구의 안정성(stability)과 관련된 신뢰도를 반영하는 지표로 내용타당도 검증 사용에 적합하지 않을 뿐 아니라, 측정하고자 하는 구성개념과 관련성이 없는(1점 또는 2점) 것도 함께 포함되어 계산되는 것이 문제라고 하였다[16]. 이에 대해 대안으로, 관련성이 낮은 문항(1점 또는 2점의 문항)을 제외한 수정된 카파계수 [“κ*=(I - CVI - P_c_)/(1 - P_c_)”가 제시되었다. 수식에서 P_c_는 N!/A!(N - A)!]×5^N^으로, N은 총 전문가의 수, A는 문항의 관련성에 대해 3점 또는 4점에 응답한 수를 의미한다[16].

여러 연구자들이 내용타당도를 판단하기 위해 정량화에 노력을 기울였지만, Beckstead [17]는 정량화를 위해서는 전문가 수가 충분히 많아야 하고, 관련성에 대한 4점 척도 점수를 이분형(1–2점, 관련성이 없음; 3–4점, 관련성이 있음)으로 재구성하여 원래 가지고 있던 정보를 손실시키지 말아야 하며, 카파 또는 수정된 카파계수에는 표준오차가 같이 제시되어야 한다고 지적하였다. 더 나아가 “타당도”란 연구자가 실제 대상자로부터 수집한 반응점수를 가지고 추론하는 것이기 때문에 문항 자체에 대한 전문가 판단 결과에 타당도라는 용어를 사용하는 것은 적합하지 않다고 하였다. 따라서 내용타당도보다는 “조작적 정의에 대한 수용성(acceptability of an operational definition)”이라고 명명하는 것이 바람직하다고 하였다.

위에서 제시된 내용타당도는 모두 도구 내용의 관련성에 대한 것이다. 내용타당도에는 이외에도 측정도구에 포함된 내용이 얼마나 포괄적인지, 측정도구를 사용하고자 하는 사람들이 얼마나 이해할 수 있는지에 대한 것도 같이 평가되어야 한다[6]. 많은 연구자들은 내용타당도의 관련성 평가에는 노력을 기울이지만, 포괄성이나 이해성 평가에 대해서는 간과하는 경향이 있다. 일례로 최근 5년간 한 학술지에 게재된 27편의 심리계량적 속성에 대한 고찰연구를 살펴보면, 내용타당도에서 관련성은 77%의 연구에서 평가되었던 반면, 포괄성과 이해성은 각각 3.7%, 48.2%만 실시되었다[18]. 또한 Lee 등[19]이 실시한 13개 당뇨병 자가관리 측정도구에 대한 체계적 고찰을 보면, 내용타당도의 관련성에 대한 질적 근거는 주로 보통이었지만, 포괄성 및 이해성은 대부분 낮음 또는 매우 낮음으로 나타났다. 즉, 측정도구 개발 시 내용타당도 관련성에 대한 검증에 비해 포괄성 및 이해성에 대한 고려가 상대적으로 부족했다는 것을 암시한다. 미국 식품의약국 가이드라인[2]에 의하면, 내용타당도에는 전문가뿐 아니라 측정도구 사용대상자도 포함할 것을 권고하고 있다. 따라서 사용대상자에게도 내용타당도의 관련성, 포괄성, 및 이해성이 평가되어야 할 것이다.

## 요약

내용타당도는 측정도구의 내용이 측정하고자 하는 구성개념을 얼마나 적절히 반영하고 있는가에 대한 것이다. 그 과정은 예비측정도구 개발 단계와 개발된 것이 측정하고자 하는 구성개념을 잘 반영하는지를 판단하는 단계로 나누어 볼 수 있다. 첫 번째 단계에서는 측정하고자 하는 구성개념에 대한 개념화와 예비질문지 개발이 이루어진다. 그 후, 전문가와 측정도구를 사용하고자 하는 대상자를 대상으로 개발된 예비문항의 관련성, 포괄성, 및 이해성을 판단하게 된다.

## References

[1] Stevens SS (1951). Handbook of experimental psychology.

[2] U.S. Food and Drug Administration (2009). Guidance for industry on patient-reported outcome measures: use in medical product development to support labeling claims. Fed Reg.

[3] Mokkink LB, Terwee CB, Patrick DL, Alonso J, Stratford PW, Knol DL (2010). The COSMIN study reached international consensus on taxonomy, terminology, and definitions of measurement properties for health-related patient-reported outcomes. J Clin Epidemiol.

[4] Lynn MR (1986). Determination and quantification of content validity. Nurs Res.

[5] Polit DF, Yang FM (2016). Measurement and the measurement of change.

[6] Terwee CB, Prinsen CA, Chiarotto A, Westerman MJ, Patrick DL, Alonso J (2018). COSMIN methodology for evaluating the content validity of patient-reported outcome measures: a Delphi study. Qual Life Res.

[7] Lee HJ, Song R, Lee EH, Ann SH (2017). Research methods and critical appraisal.

[8] Lee EH, Tahk SJ, Shin JH, Lee YW, Song R (2007). Development and psychometric evaluation of cardiovascular disease-specific quality of life scale for Koreans. J Korean Acad Nurs.

[9] Yun YH, Park YS, Lee ES, Bang SM, Heo DS, Park SY (2004). Validation of the Korean version of the EORTC QLQ-C30. Qual Life Res.

[10] Lee EH, Chun M, Wang HJ, Lim HY, Choi JH (2005). Multidimensional constructs of the EORTC Quality of Life Questionnaire (QLQ-c30) in Korean cancer patients with heterogeneous diagnoses. Cancer Res Treat.

[11] Jung YM, Park JH (2017). Development and validation of the cancer-specific posttraumatic growth inventory. J Korean Acad Nurs.

[13] Lawshe CH (1975). A quantitative approach to content validity. Pers Psychol.

[14] Polit DF, Beck CT (2006). The content validity index: are you sure you know what’s being reported? Critique and recommendations. Res Nurs Health.

[15] Wynd CA, Schmidt B, Schaefer MA (2003). Two quantitative approaches for estimating content validity. West J Nurs Res.

[16] Polit DF, Beck CT, Owen SV (2007). Is the CVI an acceptable indicator of content validity? Appraisal and recommendations. Res Nurs Health.

[17] Beckstead JW (2009). Content validity is naught. Int J Nurs Stud.

[18] Lee EH, Kang EH, Kang HJ (2020). Evaluation of studies on the measurement properties of self-reported instruments. Asian Nurs Res.

[19] Lee J, Lee EH, Chae D, Kim CJ (2020). Patient-reported outcome measures for diabetes self-care: A systematic review of measurement properties. Int J Nurs Stud.

